# Evaluation of salivary placental growth factor in Health and Periodontitis

**DOI:** 10.1186/s12903-024-04282-x

**Published:** 2024-04-26

**Authors:** Maryam Humaid Aljarwan Alshamsi, Aghila Rani Koippallil Gopalakrishnan, Betul Rahman, Anirudh B. Acharya

**Affiliations:** 1 Dubai Health Authority Center, Dubai, UAE; 2 Sharjah Institute for Medical Research, University of Sharjah, Sharjah, UAE; 3 Department of Preventive and Restorative Dentistry, College of Dental Medicine, University of Sharjah, Sharjah, UAE

**Keywords:** Placental growth factor, Periodontitis, Health, Saliva, ELISA

## Abstract

**Background:**

Various immune mediators have a role in the progression of periodontitis. Placental Growth Factor (PLGF) is important during pregnancy and also is involved in the pathology of several diseases. Hence, this study aimed to evaluate salivary PLGF in health and periodontitis that seemingly has not been reported earlier.

**Methods:**

Fifty participants were grouped as healthy and periodontitis patients. Clinical history, periodontal parameters [Plaque Index (PI), Gingival Index (GI), probing pocket depth (PPD), clinical attachment loss (CAL), bleeding on probing (BoP)] were recorded; saliva was collected and PLGF was estimated using a commercially available ELISA kit. The data were statistically analyzed using Shapiro-Wilk’s test, Kruskal-Wallis test, Dunn’s post hoc test with Bonferroni correction, and Spearman’s rank-order correlation coefficient. The significance level was set at *p* ≤ 0.05 for all tests.

**Results:**

Salivary PLGF levels comparison between the two groups showed no significant difference between both groups. Quantitatively, females had higher salivary PLGF levels than males. No significant association was observed between salivary PLGF levels and the severity of periodontitis. The periodontitis group showed statistically significant correlations between salivary PLGF levels, BoP(*p* = 0.005) and PPD(*p* = 0.005), and significant correlations of PLGF with PPD (*p* = 0.035) for both groups.

**Conclusions:**

PLGF can be detected and measured in the saliva of healthy individuals and periodontitis patients. However, the role of PLGF in periodontal pathology needs to be further confirmed based on their salivary levels.

## Background

Periodontal pathology, that mainly include gingivitis and periodontitis are the inflammatory activities/processes that involve the periodontium, i.e., the tooth supporting structures. The current model of pathogenesis of periodontal diseases underlines the complex interactions among plaque (oral biofilm) bacteria, the host’s genetic factors, and acquired environmental stressors [1].

Inflammatory mediators have captivated much attention as potentially crucial variants influencing the host response in periodontitis. Host-derived cytokines for example, released upon microbial challenge have significant effects on the immune and inflammatory responses in periodontal diseases [2, 3]. Several cytokines have a recognized role in the pathogenesis of periodontal disease including interleukin-1(IL-1), interleukin-6 (IL-6), tumor necrosis factor- α (TNF- α) and vascular endothelial growth factor (VEGF). Several members of VEGF family have been described as VEGF-A to E along with placental growth factor (PLGF) [4–6].

PLGF is a homodimeric glycoprotein that is encoded by a single gene which generates four isoforms that differ in the size and binding properties: PLGF-1, PLGF-2, PLGF-3, and PLGF-4. PLGF-1 and PLGF-2 are the common ones. It was originally identified in human placenta by Persico in 1991 [7], following the discovery of two members of VEGF; it is responsible for support of trophoblasts growth and differentiation [7, 8]. PLGF is found in various organs and tissues in pathological process involving heart, thyroids, lungs and muscles, in adulthood [9]. In addition to that, many cells release this growth factor upon stimulation: endothelial cells, vascular smooth muscle cells, leukocytes, bronchial epithelial cells, fibroblast, bone marrow cells, neurons, keratinocytes, and many tumor cells [10].

Though substantial biomarker research has been undertaken to comprehend the origination and development of periodontitis, several markers of inflammation are yet to be analyzed to demonstrate the sophisticated interconnections at the molecular state. Therefore, additional explorations are needed to elucidate the role and contribution of less investigated molecular biomarkers. One of the known important biological molecules is PLGF that is closely connected to placental or maternal biological activity and may have a role in periodontal health and disease.

The body fluids like saliva, gingival crevicular fluid (GCF), serum, plasma amongst others, are sources of biomarkers. Saliva is one of the definitive biological fluids for the investigation of biomarkers especially for their detection and estimation in oral diseases. Saliva contains proteins, enzymes, microbes and its products, several growth factors and genetic material. Saliva is being considered as an ultra-filtrate of serum, thus reflecting normal physiological and pathological process like serum. Moreover, there are several benefits of using saliva for the diagnostic purpose as it is convenient to use, non-invasive, inexpensive, effortless to acquire as compared to other body fluids. Hence, saliva was considered for this study and not gingival crevicular fluid. Multiple sampling, detection of several biomarkers from one sample and having real-time diagnostic value are additional benefits of saliva. Furthermore, there are swift advances in salivomics and commercially available kits for biomarkers detection [11, 12]. The behavior of PLGF in periodontitis is less known. Studies pertaining to the same will help in understanding its role in periodontal disease. The objectives of this study were evaluation of salivary PLGF in health and periodontitis and to investigate the potential role of PLGF in the pathogenesis of periodontal disease and correlation of level of PLGF with severity of inflammation, severity of periodontitis. To the best of knowledge, there are no studies about salivary PLGF levels and its relationship to periodontitis in the literature.

## Methods

This study was conducted in the College of Dental Medicine and University Dental Hospital Sharjah of the University of Sharjah, Sharjah, UAE. An ethical clearance was obtained from the institutional ethical committee (REC-21-09-28-01-S) and informed written consent was obtained from all the participants, and participant information sheet was provided to each, prior to their enrolment in the study. All the clinical procedures were performed in full accordance with the revised Declaration of Helsinki and the Good Clinical Practice Guidelines.

### Study sample

This cross-sectional study included 50 participants, who visited the clinics of the University Dental Hospital Sharjah, and the Periodontology Post Graduate Clinics at the College of Dental Medicine, University of Sharjah, Sharjah. The participants were categorized as Group 1 (systemically and periodontally healthy) and Group 2 (periodontitis patients who are systemically healthy).

### Sample size calculation

As there were no studies in the literature for reference regarding this current study, it was estimated that about 24 subjects with periodontitis would be required for 90% power, assuming α = 0.05, δ = 0.6%, and σ = 1.0%. With an estimated 50% prevalence of such individuals in the cohort, about 48 participants were required to be recruited as per the selection criteria.

### Inclusion and exclusion criteria

The inclusion criteria were participants with at least 20 natural teeth present, in the age range of 20–55 years and having generalized periodontitis {generalized periodontitis is 30% or more teeth involved, stage II (moderate); stage III and IV (severe) based on the 2017 classification [13, 14], probing pocket depth (PPD) of ≥ 3 to 4 mm, clinical attachment loss (CAL) of ≥ 3 mm, which is positive for bleeding on probing (BoP) and with radiographic evidence of bone loss}. Female participants who were never pregnant or have past 5 years since delivery of their baby were included. The exclusion criteria were individuals with any systemic diseases/disorders, the presence of any disease that may alter the immune system/immunocompromised individuals, individuals who are currently on antibiotics or having used them in the past three months and/or anti-inflammatory drug regimen, pregnant, lactating, menopausal women, tobacco smokers and/or tobacco chewers/users, and any periodontal treatment for the last 3 months.

### Study groups

The 50 study volunteers were grouped equally as Group 1 comprising 25 systemically healthy participants and Group 2 comprising 25 systemically healthy participants who had generalized moderate-severe periodontitis. Participants were selected for each group after a thorough and precise case history recording that included patient’s chief complaint, clinical examination and evaluation. The same operator carried out clinical evaluation and measurements after intra-examiner calibration (kappa statistic value of 0.83, 94% agreement). The participants were seated comfortably in an upright position on a dental chair and the area to be examined was well illuminated. Appropriate armamentarium was used to clinically examine the periodontal status using the UNC-15 periodontal probe. Periodontal parameters assessed included Plaque Index (PI) [15], Gingival Index (GI) [16], PPD, CAL and BoP.

### Collection of Saliva

The saliva was collected based on previously reported literature [17]. Before the collection of saliva, the study subjects were instructed to not consume foods and beverages with high sugar/acid/caffeine content, no alcohol intake or use any medications/mouthwash (for at least 12 h before saliva collection), not to eat a major meal (within 1 h of saliva collection), not to brush teeth (for at least 45 min before saliva collection), no dental work should have been done on them in the past 24 h, as this would compromise the assay. Unstimulated saliva samples were collected at uniform times of the day from each of the participants after rinsing the mouth with saline for 30 s and then were requested to expectorate in the sink to remove any residues and wait for at least 10 min after rinsing to avoid dilution of the sample before collecting saliva. The samples were centrifuged at 2500 rpm for 10 min to remove particulate debris and separation of the fluid phase for analysis. A hundred µl of the fluid phase was used for the experiments.

#### ELISA

Salivary PLGF was assessed in Group 1 and Group 2 using- PLGF Human ELISA Kit [18] (Abcam PLC, Cambridge, United Kingdom, Catalogue No. ab100629; range: 1.372–1000 pg/mL; sensitivity < 2 pg/mL). The ELISA protocol was followed as per the manufacturer’s instructions to estimate the protein from the saliva samples.

### Data Analysis

Categorical data were presented as frequencies and percentages and were analyzed using the chi-square test. Numerical data were presented as mean and standard deviation values. They were explored for normality using Shapiro-Wilk’s test. Data were non-parametric and were analyzed using the Kruskal-Wallis test followed by Dunn’s post hoc test with Bonferroni correction. Correlations were analyzed using Spearman’s rank-order correlation coefficient. The significance level was set at *p* ≤ 0.05 for all tests. Statistical analysis was performed with R statistical analysis software version 4.1.3 for Windows.

## Results

### Demographic data and measured parameters

Demographic data and the measurements of parameters are presented in Table 1. The study was conducted on 50 participants (i.e., 25 per group). There were 18 males (mean age 38.83 years) and 7 females(mean age 38.85 years) in Group 2 (9 stage II, 14 stage III and 2 stage IV periodontitis patients, respectively), while in Group 1 there were 13 males(mean age 28.15 years) and 12 females(mean age 29.75 years). The difference between both groups was not statistically significant. Group 2 had a significantly higher age than Group 1. The mean PLGF was higher in the healthy group as compared with the periodontitis group. There was no significant difference between both groups.

**Table 1:** Demographic data and measured parameters

Parameter			Group 2 (Periodontitis)	Group 1 (Health)	*p*-value
Sex	Male	n	18	13	0.244
		%	72.0%	52.0%	
	Female	n	7	12	
		%	28.0%	48.0%	
Age (years)	Mean ± SD		38.52 ± 8.99	28.92 ± 9.25	< 0.001*
PI	Mean ± SD		86.92 ± 8.75	27.20 ± 5.71	< 0.001*
BOP	Mean ± SD		86.32 ± 8.34	7.32 ± 1.84	< 0.001*
GI	Mean ± SD		1.71 ± 0.34	0.01 ± 0.02	< 0.001*
PPD	Mean ± SD		5.33 ± 0.62	2.12 ± 0.30	< 0.001*
CAL	Mean ± SD		5.11 ± 0.69	0.01 ± 0.01	< 0.001*
PLGF (in pg/ml)	Mean ± SD		85.87 ± 23.54	87.90 ± 23.09	0.473

* Statistically significant

### Association between gender and PLGF level

The association between gender and PLGF level is presented in Table 2. For both groups and overall, females had a higher mean value, yet the difference between both genders was not statistically significant (*p* > 0.05). Additionally, within each gender, there was no significant difference between both groups (*p* > 0.05).

**Table 2:** Association between gender and PLGF levels

Group	PLGF (Mean ± SD)		*p*-value
	Male	Female	
Group 2 (Periodontitis)	82.19 ± 23.69	95.35 ± 21.93	0.155
Group 1 (Health)	82.90 ± 19.93	93.32 ± 25.87	0.384
*p*-value	0.368	0.767	
Overall	82.49 ± 21.84	94.07 ± 23.88	0.056

* Statistically significant

### Association between periodontitis severity and PLGF level

The association between periodontitis severity and PLGF levels is presented in Table 3. The association was not statistically significant, and there was no difference in the PLGF concentrations between the periodontitis patients with stage III and stage IV periodontitis.

**Table 3:** Association between periodontitis severity and PLGF levels

PLGF (Mean ± SD)			*p*-value
Healthy	Moderate periodontitis	Severe periodontitis	
87.90 ± 23.09	94.15 ± 25.50	82.65 ± 22.67	0.384

* Statistically significant

### Correlations between different periodontal parameters and PLGF levels

**Fig. 1:**
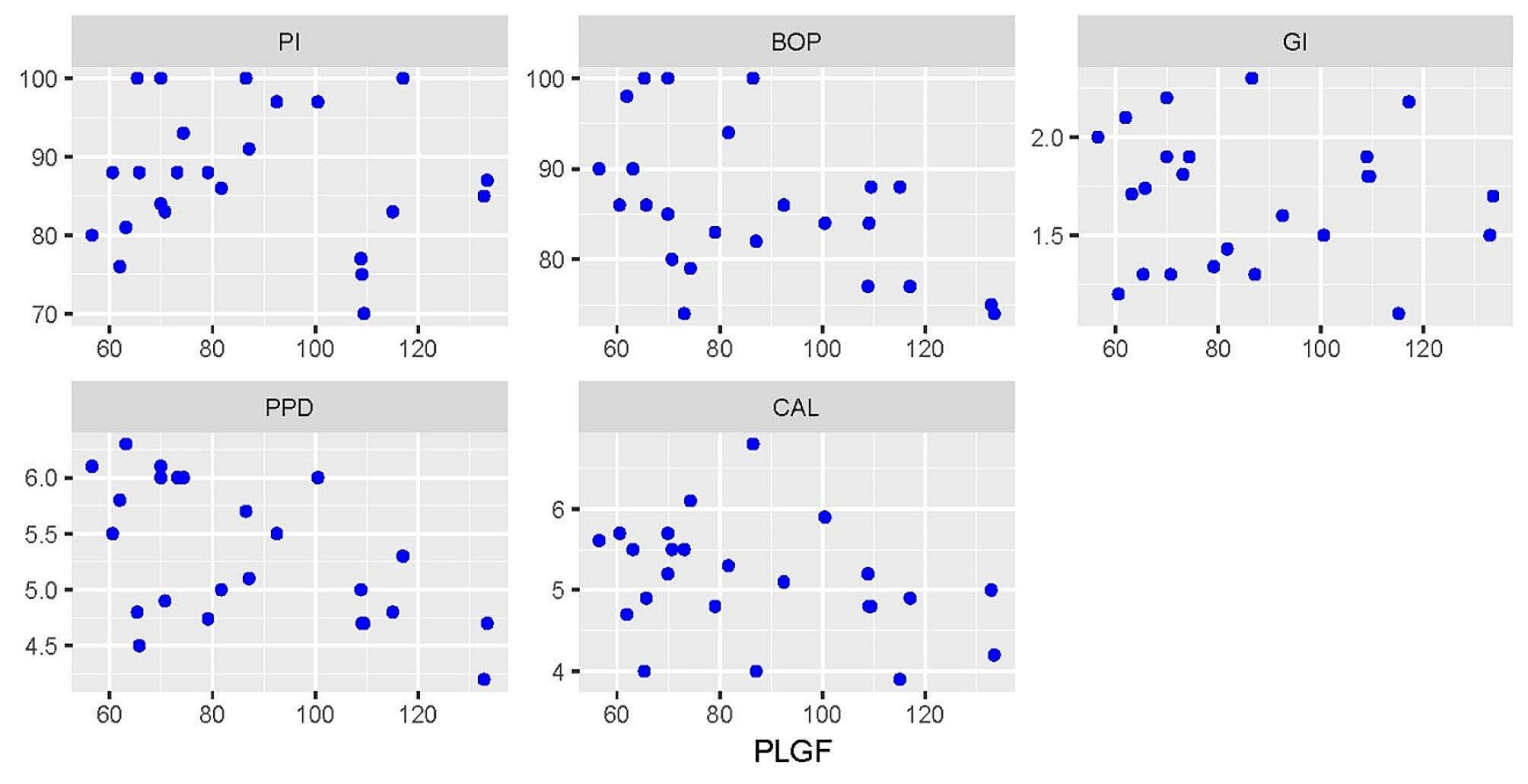
Scatter plot showing the correlations between different periodontal parameters and PLGF level in the periodontitis group

**Fig. 2:**
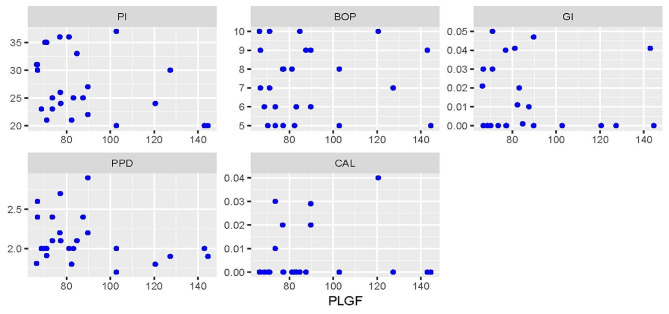
Scatter plot showing the correlations between different periodontal parameters and PLGF level in the healthy group

**Fig. 3:**
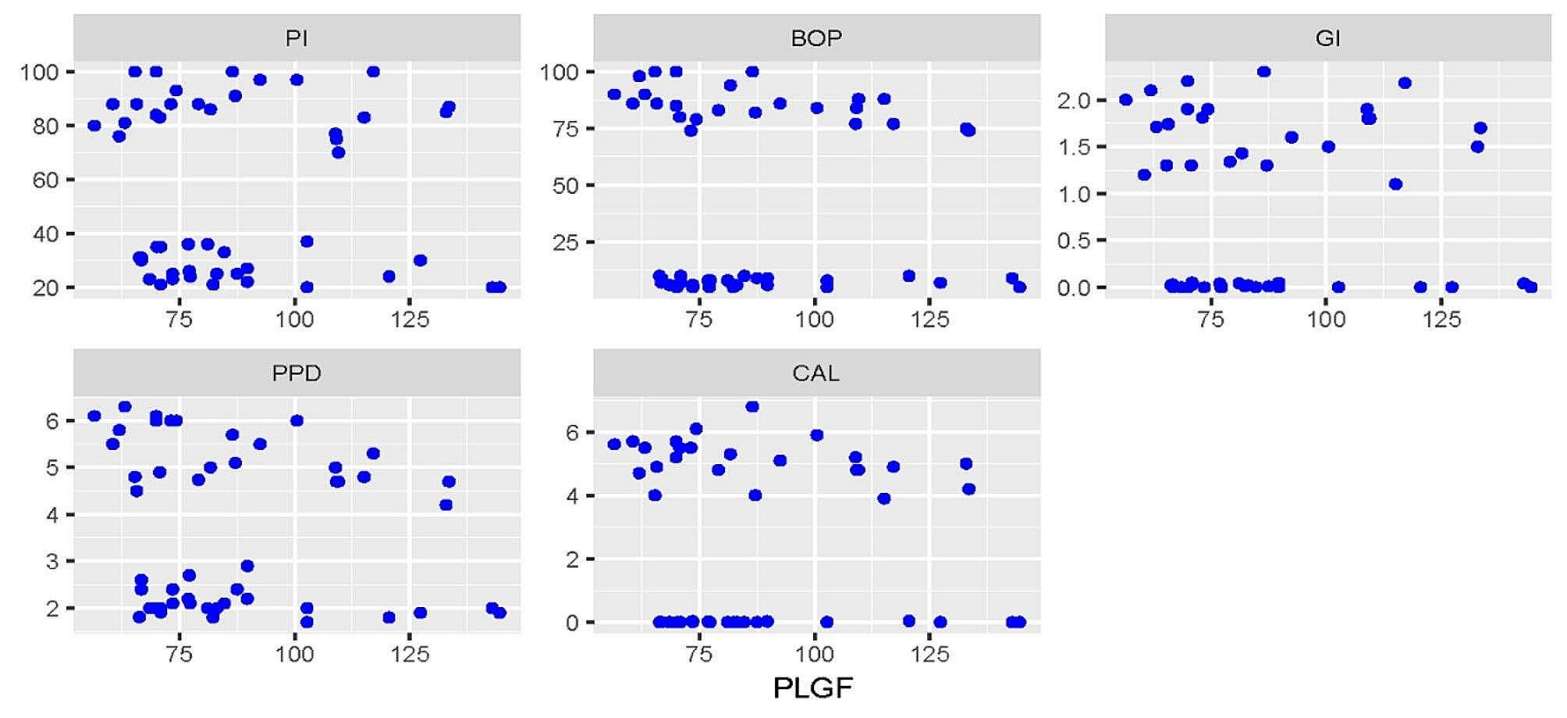
Scatter plot showing the correlations between different periodontal parameters and PLGF levels in both groups

Correlations between different periodontal parameters and PLGF levels are presented in Figs. 1, 2 and 3. For the periodontitis group, there were statistically significant negative correlations with BOP (rs=-0.540, *p* = 0.005) and PPD (rs=-0.550, *p* = 0.005). For both groups, there was a negative correlation with PPD which was statistically significant (rs=-0.300, *p* = 0.035). All other correlations were not statistically significant (*p* > 0.05).

## Discussion

The objective of the research was to analyze and compare the PLGF in the saliva of healthy individuals and periodontitis patients, in general, and investigating the role of PLGF in the pathogenesis of periodontitis and correlating PLGF’s levels with the severity of periodontitis, in specific. PLGF in saliva was detected and quantified in all the samples that were subjected to analysis, and there was a quantifiable difference between health and periodontitis.

Several biomarkers and their biological actions in periodontal pathology have been extensively discussed in the literature. However, the interconnections among these significant biological molecules, signaling pathways, and reciprocations are not entirely comprehensible yet. This has led to assessing the role of these markers of inflammation in the periodontal tissues, GCF, saliva and serum. Various biomarkers have been extensively analyzed in health and disease and a quantifiable difference in most of the evaluated biological molecules has been reported [19–21].

An Italian scientist Dr. Maria Graziella Persico in 1991, first reported PLGF after its discovery. It was recognized when analyzing the angiogenic capacity of the human placenta, hence this biological molecule was named “the placental growth factor” [7]. As described earlier, PLGF is representative of the VEGF family that also includes the structurally and functionally connected angiogenic factors VEGF-A to VEGF-E. Although identified first in the placenta, PLGF is more than a specific maternal biomarker. PLGF expression is high during the development of the fetus in the placenta, heart, lung, thyroid, brain, skeleton muscle, and gingival epithelium but lowers in adulthood. PLGF is also expressed in the heart, lung, thyroid, adipose tissue, and skeletal muscle of adults [10]. Whether PLGF is a potential biomarker of disease or has a role in targeted vascular disease therapies remains the focus of continuing research. It has been reported that PLGF is present in human oral fluids and is a biomarker for preeclampsia and gestational diabetes mellitus [22]. Although PLGF is discussed in relation to several inflammatory conditions as atherosclerosis and rheumatoid arthritis, to the best of our knowledge the present study may be the first that is examining salivary PLGF levels and periodontitis.

In this study, salivary PLGF levels were compared in periodontal health and in cases of moderate or severe periodontitis with the consideration that concentrations of unstimulated saliva analytes are not affected by diurnal salivary flow rate as per Carpenter [23]. The results were not statistically significant with regard to PLGF levels in health and periodontitis. Also, no significant correlation between the severity of the periodontitis and the levels of PLGF were noted, except for a statistically significant negative correlation of PLGF levels with PPD and BOP, that has not been reported earlier, leading to the possibility of PLGF to be quantitatively lower in a local inflammatory condition such as periodontitis. It could offer the explanation that PLGF may be protective instead of pro-inflammatory as reported in maternal conditions. In another study [24], it was found that reduced PLGF activity is involved in anti-inflammatory effects.

Sert et al. [5], evaluated the levels of serum IL-1β, IL-6, TNF-α, IL-10, VEGF, PLGF, and sVEGFR-1, and − 2 in the association between periodontal disease and adverse pregnancy outcomes; according to their results, there were no significant differences in the serum PLGF level between health, gingivitis, and periodontitis, which is also pointing at the inconsistencies of the role of PLGF for a definitive conclusion. Conversely, Chaparro et al. [18]. , in a study on maternal oral fluids (i.e. saliva and GCF) concentrations of PLGF and sFlt-1 in conjunction with periodontal inflammatory status early in pregnancy (11 to 14 gestation weeks) found that maternal PLGF concentrations in GCF were significantly increased in pregnant women with periodontitis who later develop GDM, while PLGF concentrations in saliva did not show statistically significant differences which is similar to our results, and they emphasized on clarification of the role of placental mediators in periodontal tissues. Tseng et al. [25] have reported an upregulation of PLGF and Leblebicioglu et al. [26]. , , observed that PLGF levels of GCF increase in wounding and inflammation. Our results indicate salivary PLGF to be lower in periodontitis as compared with health. In the context of our study all these reports about pregnant women open up further challenges to interpret our observations of PLGF levels in saliva independent of pregnancy, as to whether to attribute a pro-, or anti-inflammatory behavior with regard to periodontitis.

We also assessed the PLGF level in relation to gender (the presence of PLGF in saliva was confirmed in both males and females); females had a higher mean value yet the difference between both genders was not statistically significant (*p* > 0.05). Additionally, there was no significant difference between groups 1 and 2 (*p* > 0.05) in terms of a specific gender. Although none of the previous studies in the literature have mentioned this association, it can be implied that PLGF can be detected in both genders (not necessarily only in females during pregnancy).

The current investigation has accomplished the general objective of analyzing and comparing PLGF in the saliva of health and periodontitis and a quantifiable difference between these two groups was noted, though not statistically significant. Specifically, the role of PLGF in the pathogenesis of periodontitis is not conclusive. Regarding the correlation of PLGF levels with periodontitis, it was observed that PLGF might be potentially anti-inflammatory in action, viewing its relationship with two significant variables, i.e., BOP and PPD. If the biologic behavior of PLGF is comparable to VEGF, then it is plausible that in periodontitis, PLGF may not be released as it may be bound to tissue. Clarification of PLGF activity with respect to periodontitis needs more research.

## Conclusions

Saliva is a reliable source for the detection and estimation of PLGF. PLGF should be investigated further by using other sources such as gingival crevicular fluid or serum to explain its potential role in periodontitis. The present investigation has limitations of being a cross-sectional design that makes generalizing the findings and interpretation of results difficult. More longitudinal studies should be undertaken in the future that may help in providing clarity on the role of PLGF in periodontitis.

## Data Availability

The datasets used and/or analyzed during the current study are available from the corresponding author on reasonable request.

## Abbreviations

SD: Standard deviation
PI: Plaque Index
BOP: Bleeding on probing
GI: Gingival Index
PPD: Probing pocket depth
CAL: Clinical attachment loss
PLGF: Placental Growth Factor

## Declarations

All experimental protocols were approved by the research ethics committee of the university of Sharjah.
