# Correction: Evaluation of salivary placental growth factor in Health and Periodontitis

**DOI:** 10.1186/s12903-024-04629-4

**Published:** 2024-07-28

**Authors:** Maryam Humaid Aljarwan Alshamsi, Aghila Rani Koippallil Gopalakrishnan, Betul Rahman, Anirudh B. Acharya

**Affiliations:** 1 Dubai Health Authority Center, Dubai, UAE; 2 Sharjah Institute for Medical Research, University of Sharjah, Sharjah, UAE; 3 Department of Preventive and Restorative Dentistry, College of Dental Medicine, University of Sharjah, Sharjah, UAE

**Correction to: BMC Oral Health (2024) 24:493**

10.1186/s12903-024-04282-x

The Acknowledgements section was missing from this article [1], and should have read as below.
